# Quality of seminal fluids varies with type of stimulus at ejaculation

**DOI:** 10.1038/srep44339

**Published:** 2017-03-13

**Authors:** E. Jeannerat, F. Janett, H. Sieme, C. Wedekind, D. Burger

**Affiliations:** 1 Swiss Institute of Equine Medicine ISME, Agroscope and University of Berne, Avenches, Switzerland; 2 Clinic of Reproductive Medicine, University of Zurich, Zurich, Switzerland; 3 Unit for Reproductive Medicine – Clinic for Horses, University of Veterinary Medicine Hanover, Hanover, Germany; 4 Department of Ecology and Evolution, Biophore, University of Lausanne, Lausanne, Switzerland

## Abstract

The theory of ejaculate economics was mainly built around different sperm competition scenarios but also predicts that investments into ejaculates depend on female fecundity. Previous tests of this prediction focused on invertebrates and lower vertebrate, and on species with high female reproductive potential. It remains unclear whether the prediction also holds for polygynous mammals with low female reproductive potential (due to low litter size and long inter-birth intervals). We used horses (*Equus caballus*) to experimentally test whether semen characteristics are adjusted to the oestrous cycle of the mare a stallion is exposed to during few moments before ejaculation. We analysed 122 weekly semen samples collected from 16 stallions during exposure to either an oestrous or a dioestrous mare. Semen volume and the rate of motile sperm were higher when stallions were exposed to an oestrous than to a diestrous mare, while total sperm counts and sperm velocity remained unchanged. Sperm collected after exposure to an oestrous mare also showed reduced oxidative degeneration of cell membranes over a period of 48 hours. We conclude that stallions invest more into their seminal fluids when the chance of fertilization is elevated, and that this adjustment of ejaculate quality can happen very quickly.

The theory of ejaculate economics^1^ makes strong prediction about the evolution of reproductive traits and about phenotypic plasticity in male reproductive parameters. Many of these predictions have successfully been tested in comparative studies and in experimental work within species. For example, testes differ morphologically and ejaculates generally contain more sperm in species or populations with more sperm competition^2^,^3^,^4^, and males can quickly adjust their investment into an ejaculate to the expected level of sperm competition^5^,^6^,^7^,^8^. A general conclusion from this theoretical and empirical work is that sperm competition favours large and high-quality ejaculates, and, because the possibility of sperm competition is probably ubiquitous, costs and benefits of ejaculates therefore need to be optimized. These costs and benefits are predicted to not only depend on the number of rival males but also on female quality. Males are expected to differentially invest into ejaculate quality if females vary in fecundity^1^,^9^.

Regardless of whether an ejaculate is adjusted to the number of rivals or the fecundity of a female, an important question in ejaculate economics is always whether each sperm has the same chance of fertilizing an egg^1^, i.e. whether the raffle is “fair” or “loaded”. Reasons for a loaded raffle can be extrinsic, including differences in the timing of insemination (e.g. relative to the timing of oestrus), or intrinsic, due to differences in sperm quality or the quality of seminal fluids. Key factors in ejaculate economics are therefore not only sperm number per ejaculate but also sperm velocity and sperm longevity. Arguably, variation in sperm velocity may be more relevant in external fertilizers where fertilization happens within seconds and sperm longevity is typically in the range of few seconds or minutes^10^, while variation in sperm longevity may be more relevant in internal fertilizers where sperm may need to remain functional for longer times to successfully fertilize an ovum (e.g. several hours or days in many mammals, including humans and horses). In the latter case, the ability of seminal fluids to support sperm longevity (e.g. to protect them from oxidative damage) may play a decisive rule^11^.

Seminal fluids often make up a larger part of an ejaculate. They may contain specific proteins, hormones, antimicrobials, immunity suppressors, fats, sugars, salts, and different types of cells, including immunity cells^12^,^13^. The composition of seminal fluids is generally expected to vary with male genetics, condition, life history, and social situation^13^, with different functions for different components^14^,^15^. Little is known about the relative cost of the various components. However, seminal fluids have important effects on female reproductive physiology^16^ and on male and female fitness^17^, and the availability of seminal fluids has been found to limit remating^18^. Males are therefore predicted to rapidly modify the composition of ejaculates in response to female characteristics. Such a rapid adjustment may be under hormonal control, e.g. by testosterone that can rise quickly in response to the presence of a female^19^ and that has been found to influence characteristics of seminal fluids^20^,^21^.

We chose the polygamous horse (*Equus caballus*) to test experimentally whether blood testosterone concentration, behavioural indicators of sexual arousal, and ejaculate characteristics vary with oestrous cycle of a stimulus mare that a stallion is exposed to shortly before and during ejaculation. We used a within-subject experimental design to control for possible variance among stallions. Semen quality was determined immediately after ejaculation and over a period of 48 hours thereafter.

## Material and Methods

Sixteen clinically healthy and sexually experienced stallions (mean age = 12.1 years, range 5–19) and mares A and B (11 and 13 years old, respectively, with regular oestrous cycles) were used for the experiments (all of Franches-Montagnes breed and unrelated to each other). They were kept in individual boxes bedded with straw, with other horses of only the same sex in the same stable each, i.e. the sexes were separated in different stables and there was no contact between the sexes other than during experimental exposure. All horses were regularly exercised and had daily access to a paddock. The study was approved by the *Etat de Vaud, Service Vétérinaire* (permit no 2667.1). All methods were performed in accordance with the relevant guidelines and regulations (Vaud canton and Swiss Confederation).

The experiments lasted over a period of 7 weeks and took place every Tuesday in a building that was separated from the housing stables. One of the two mares was led to this building and tethered close to a dummy. Stallions were then individually led to this building where they were first exposed to this mare’s faeces for 15 seconds (presented on a plastic plate on the ground). The stallion was then held in front of the mare’s head at a distance of about 2 meters and for about 30 seconds, then led to a dummy that the stallion could mount and that was used to collect the semen in an artificial vagina (type “Avenches”). A second faeces sample of the stimulus mare was hung in a perforated bag above the dummy. The stallion was then returned to its box, the hind part of the dummy was wrapped with a new plastic film (routine sanitary procedure), and the procedure was repeated with the next stallion. After 8 stallions, the mare was replaced by the other one, and the experiments continued with the remaining 8 stallions and with new faeces samples from this other mare. In order to ensure a crossover study design, the order of presentation changed over the first 6 weeks so that by the end of the 7^th^ week, each stallion had been exposed to each mare once in oestrus and 2 or 3 times in dioestrus. The stallions were always handled by the same person who was not informed about the study design and its objectives.

The cycles of the two mares were regularly assessed by rectal ultrasonography of ovaries and uterus. The mares were in dioestrus on two or three consecutive Tuesdays and in oestrus the following Tuesday. Oestrus was defined as existence of a follicle >35 mm, uterus oedema at stage >2^22^ combined with typical oestrous behaviours that include “sawhorse” posturing with lowered pelvis, vocalizations, frequent urination, and rhythmic eversion of the clitoris^23^,^24^. Oestrous mares were given 1500 IE hCG (Chorulon^®^, Intervet, Boxmeer, Netherlands) intravenously on the Monday evening before the experimental exposures on the following Tuesday in order to induce ovulation.

The behaviour of both the mare and the stallion was filmed (two cameras from different angles) to record time until erection, first mount on the dummy, and ejaculation confirmed by rhythmic tail flagging^25^,^26^. Mare vocalisation was recorded by an observer who did not know the oestrous cycle of the mare. Blood samples were obtained by jugular venipuncture from stallions 15 minutes before semen collection and immediately afterwards. Plasma testosterone concentrations were determined via electrochemiluminescence immunoassay (Elecsys 2010, Roche Diagnostics, Basel, Switzerland) as described previously^27^ (inter- and intra-assay coefficients of variation were 2.2 and 1.4%, respectively). Volume of the fresh ejaculate was measured after removal of the gel fraction. Sperm concentration was assessed with the Nucleocounter^®^ SP-100TM system (ChemoMetec A/S, Allerød, Denmark). Semen was then diluted with INRA 96^TM^ (IMV technologies, L’Aîgle, France) to a concentration of 30 × 10^6 ^spermatozoa/ml. Diluted semen was placed in a pre-warmed 20 μm standard count analysis chamber (Standard Count Analysis Chambers SC 20-01-C, Leja, Nieuw-Vennep, The Netherlands) and assessed for motility parameters in 10 fields using a computer-assisted sperm analyser (HTM-IVOS, version 12.1, Hamilton Thorne Biosciences, Beverly, USA) and standardized settings for stallion semen^28^. In each sample, the total motility (%), the average path velocity (VAP μm/s, average velocity of the smoothed cell path), the curvilinear path velocity (VCL μm/s, average velocity measured over the actual point-to-point track followed by the cell), and the straight-line velocity (VSL μm/s, average velocity measured in a straight line from the beginning to the end of track) of sperm cells were determined. A sample of fresh diluted semen was then packaged in an Equitainer^TM^ (Hamilton Thorne Biosciences, Beverly, USA) and sent to the Clinic of Reproductive Medicine, University of Zurich/Switzerland, for analysis of sperm motility, viability and membrane lipid peroxidation after 24 and 48 hours of cold (4 °C) storage. Evaluation of the plasma membrane and acrosome integrity as well as of the lipid peroxidation of spermatozoa was performed using a Cell Lab Quanta SC MPL flow cytometer operated by the Cell Lab Quanta SC Software for instrument Control Data Acquisition (Beckman Coulter Inc., Nyon, Switzerland). The system was equipped with a solid state LASER exciting at 488 nm and emission filters detecting green, orange and red fluorescence at 525, 590, 670 nm, respectively. Flow rate was set to 500 cells/s and for each sample 10000 events were analysed after incubation of stained sperm for 15 min at 38 °C. Membrane integrity and acrosomal status of spermatozoa were evaluated after double staining with propidium iodide (PI) and peanut agglutinin conjugated with fluorescein isothiocyanate (FITC-PNA)^29^,^30^. Five μl of the semen previously diluted with 238.5 μl Tyrode’s solution to a final concentration of 0.6 × 10^6 ^spermatozoa/ml were stained by adding 1.5 μl of 2,99-mM PI (Sigma-Aldrich, Buchs, Switzerland) and 5 μl FITC-PNA (100 μg/ml) (Sigma-Aldrich Buchs, Switzerland). The percentage of membrane and acrosome intact spermatozoa was defined as viability. For determination of lipid peroxidation in spermatozoa a combined staining with the BODIPY^581/591^-C11 probe^31^,^32^ and PI was used. 2.5 μl of 1000-μM BODIPY^581/591^-C11 (Thermo Fisher Scientific, Riehen, Switzerland) and 1.5 μl of 2,99-mM PI (Sigma-Aldrich, Buchs, Switzerland) were added to 5 μl of semen and mixed with 241 μl Tyrode’s medium to a final concentration of 0.6 × 10^6 ^spermatozoa/ml. Mean BODIPY^581/591^-C11 fluorescence intensity of live (PI negative) sperm cells was considered for further analysis.

Statistical analyses were done in JMP^®^ 11.2. Effects of stallion and mare identity (ID) on potential measures of sexual arousal and on characteristics of fresh semen were analysed in least square multiple regressions, with oestrous stage nested in mare ID. Repeated measures on consecutive testosterone measurements and on semen characteristics taken 24 h and 48 h after ejaculation were analysed in MANOVAs. Some semen and some blood samples could not be measured due to technical problems. It turned out, however, that no experimental cell of the factorial within-subject design was lost because all missed samples had been taken during exposure to dioestrous mares for which we always had several replicates per combination of stallion and mare. Therefore, using average values per stallion, time point of measurement, mare, and oestrous stage (n = 16 × 2 × 2 × 2 = 128) ensured a balanced data set for all analyses. We used directed testing (p_dir_) when there was a strong *a priori* expectancy about the direction of an effect and to avoid inflation of the alpha value^33^. If directed and two-tailed testing led to the same conclusions, two-tailed p-values (p) are reported. Kendall rank correlation coefficients τ were used to analyse correlations.

## Results

The stallions differed in mean time to erection, time to first mount the dummy, and time to ejaculation (Table 1). All these time periods were shorter when stallions were exposed to an oestrous than to a dioestrous mare (Table 1), while mare ID did not significantly influence indicators of male sexual arousal (Table 1). Both mares vocalized more intensely in reaction to a stallion and turned their head longer towards the stallion when in oestrus than when in dioestrus (mean numbers of vocalizations ±95% CI: 0.9 ± 0.5 versus 2.5 ± 1.5, paired t-tests: t = 3.5, d.f. = 15, p = 0.003; mean duration of head towards the stallion: 59.0 ± 21.2 versus 68.6 ± 10.2, paired t-tests: t = 3.3, p = 0.004), in addition to the behaviours that they only showed during oestrus such as clitoral eversion, urination and sawhorse posture.

The stallions differed in average sperm number, concentration, volume, velocity, and motility as determined immediately after semen collection (Table 1), in blood plasma testosterone levels around ejaculation, and sperm viability, velocity, motility, and lipid peroxidation 24 h and 48 h after ejaculation (Tables 2, 3). Mare ID did not seem to have an influence on semen characteristics (Tables 1, 2, 3). When the stimulus mare was in oestrus, sperm motilities were increased (Table 1), and, while sperm velocity and total sperm counts remained unchanged, sperm concentration per ejaculate had declined, i.e. overall semen volume had increased (Table 1).

**Figure 1:**
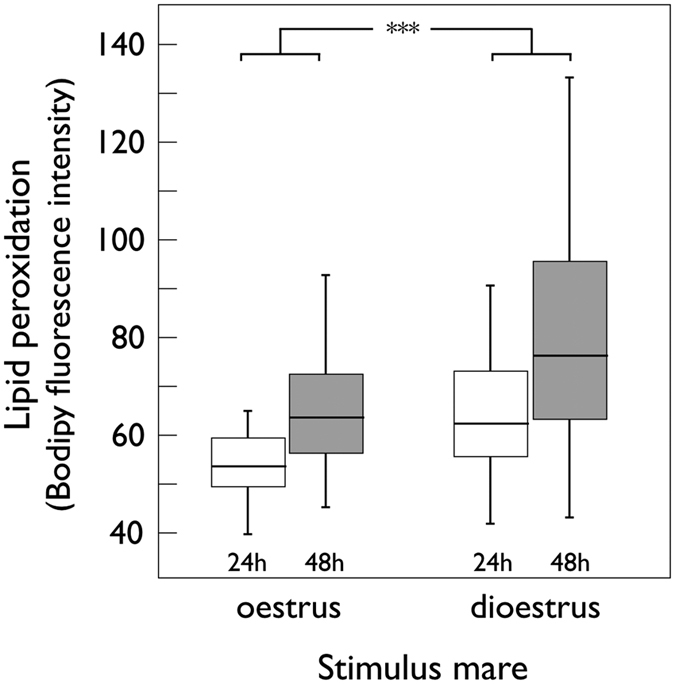
Lipid peroxidation rate (Bodipy fluorescence intensity; medians, quartiles, and deciles) of sperm membranes after 24 h (open bars) and 48 h (closed bars) when the stimulus mare was either in oestrus or dioestrus at ejaculation. See Table 2 for statistics (***p < 0.001).

Blood plasma testosterone increased from before to immediately after ejaculation (Table 2) and was not statistically linked to the oestrous stage of the stimulus mare (Table 2). However, whether the stimulus mare was in oestrus or in dioestrus had a marked effect on lipid peroxidation of sperm after 24 and 48 h cold storage (Table 2; Fig. 1). Lipid peroxidation increased and sperm viability decreased over time (Table 2; Fig. 1). Sperm velocity and motility measures taken on sperm after 24 and 48 h of cold storage were not statistically correlated to the respective lipid peroxidation measures (|τ| always ≤0.11, p always >0.29) and did not seem to be affected by the oestrus stage of the stimulus mare but were mainly linked to stallion ID and time (Table 3, see also time × stallion ID interaction effects). While the rate of motile sperm declined from 24 h to 48 h after ejaculation, the average velocity of the motile sperm increased during this observational period (Table 3). None of the observed differences in semen characteristics could be explained by stallion age or size (i.e. withers height; |τ| always <0.32, p always >0.08).

## Discussion

The stallions differed significantly in all measurements we took, from indicators of sexual arousal and testosterone blood plasma concentration to all characteristics of fresh and aging ejaculates. It therefore turned out to be important to use a within-subject experimental design that controls for this variance. The origins of such strong male effects are unclear. It has been suggested that reproductive parameters may reveal variance in health and vigour, but this hypothesis is little supported in humans^34^ (apart from several infertility aetiologies^35^ or the damaging effects of cigarette smoking and other life-style factors^36^) or in species with secondary sexual traits that may serve as quality indicators^37^. The housing conditions that our stallions experience at the stud are expected to minimize variance in health and vigour, and we found no correlations between reproductive parameters and stallion age or size. However, we cannot exclude potential effects of the order of presentation to the mares (the stallions were largely used in the same order each, and smells of previous stallions that had been tested in the experimental set up could not be avoided).

In contrast to the significant stallion effects, the ID of the two mares seemed to play no role here (however, more than two mares would be necessary to study potential mare effect). What mattered instead was whether a mare was in oestrus or not. Stallions showed behaviour that indicated higher sexual arousal when exposed to an oestrous mare and/or her faeces than when exposed to the same mares in dioestrus. The mares’ oestrous cycle also affected semen characteristics. When exposed to an oestrous mare, the stallions ejaculated more seminal fluids. Total sperm number and sperm velocity per ejaculate did not seem to increase, but sperm motility in fresh ejaculates was elevated, and sperm suffered less from oxidative stress over the following 48 hours. We conclude that the seminal fluids supported sperm motility and longevity better when the stallions were stimulated by a mare in oestrus than in dioestrus. It remains to be tested whether these effects are only due to the increased amounts of seminal fluids or also due to changes in their compositions.

It remains unclear whether testosterone played a role in inducing the effects of the mares’ oestrous cycle on semen characteristics. While blood plasma testosterone levels showed a marked increase from before to immediately after the ejaculation, we found no significant effect of the mares’ oestrous cycle on testosterone levels, neither in our between-subject not in the within-subject analyses. This suggests either that testosterone plasma levels do not react strongly to the mares’ oestrous cycle during only a short-term exposure and that the semen quality was regulated differently (for example neutrally mediated or dependent by the interaction of various hormones), or that the temporal dynamics of testosterone release requires a higher time resolution in measurements, e.g. one measurement per minute^38^, in order to reveal testosterone effects.

In fowls (*Gallus gallus*), roosters allocate larger ejaculates to more attractive hens, and seminal fluids allocated to more attractive hens support increased sperm velocity^8^. Previous studies on horses that were based on long-term exposure demonstrated that semen characteristics are plastic and that they depend on the social environments the stallions experience. Stallions showed increased sperm number per ejaculate after exposure to other stallions than after exposure to one mare only^39^, and ejaculates contained more sperm cells after exposure to mares that were predicted to be more attractive than after exposure to mares that were predicted to be less attractive^40^ (attractiveness being predicted by the sharing of alleles of the major histocompatibility complex^40^,^41^). Both conditions tested in Burger *et al*.^39^,^40^ had no or little effects on sperm velocity. These previous experiments were based on long-term exposure (4–8 weeks), covering at least the second half of the 57 days of spermatogenesis^42^. Our present experimental set-up only allowed for rapid adjustments of ejaculate characteristics. In contrast to the long-term exposure, short-term exposure seems to only affect semen volume and the ability of seminal fluids to support sperm motility and longevity, but it does not seem to affect total sperm counts. This suggests that either long-term exposure to certain social situations enhance sperm production and hence sperm number per ejaculate, or that the present treatment (oestrous versus dioestrous stimulus mare) provokes a different response than the treatments in Burger *et al*.^39^,^40^.

In feral conditions, stallions either live in harems together with several mares (and their offspring) or in bachelor bands together with other stallions. Harem stallions interact continuously with their harem mares and show signs of sexual interest (including mounting attempts) for both oestrous and dioestrous mares, even if dioestrous mares will typically try to avoid copulation^24^. Induced ovulation as in cats, rabbits, ferrets or camelids^43^ is not described for horses. However, exposure to stallion was recently found to affect the mares’ cyclicity^44^. Such potential fitness-enhancing effects may explain why stallions would try to mount dioestrous mares and ejaculate semen, even if of comparatively low quality.

Female pheromones have been found in other mammals to stimulate immediate reactions^45^. We therefore exposed the stallions to faeces samples of the mares that may reveal their oestrous stage. However, the mares were also present during semen collection, and they expressed the typical oestrous signals. It is therefore unclear what role the faeces samples played in inducing the strong treatment effects we observed here.

In horses, a typical oestrous cycle takes approximately 3 weeks during the reproductive season^44^. The typical oestrous signals can normally be observed during approximately 5–7 days during each cycle, i.e. up to several days before ovulation. Longevity of ejaculated sperm may therefore be of critical importance, and stallions who mount an oestrous mare may be expected to invest into sperm longevity, i.e. into the respective components of the seminal fluids. Mammalian seminal fluid include antioxidants like superoxide dismutase, catalase, glutathione peroxidase, and several non-enzymatic agents that protect spermatozoa from oxidative damage^13^. We found that aging sperm still suffered from increased lipid peroxidation over time, potentially contributing to the observed reduction in sperm viability over time. The oxidative stress was mitigated after exposure to an oestrous mare, i.e. seminal fluids provided increased support for sperm that were ejaculated during exposure to an oestrous stimulus mare. However, the mitigating effects of this treatment condition did not significantly affect viability, motility, or velocity of aging sperm. There was only a non-significant tendency that sperm that suffered less from lipid peroxidation also showed increased viability. Even if oxidative stress is a major cause of damage to sperm^46^, the link between lipid peroxidation and sperm viability is still not sufficiently understood^47^.

In stud farms, cooled sperm that are subject to oxidative stress are routinely used, and the per-cycle conception is typically more variable and on average clearly lower than in many other life stock^48^ (D.B. & H.S. personal observations). We found that semen quality can be significantly increased with the use of stimulus mares in oestrus. The enhanced semen quality may then provide two benefits: (i) it may increase success rates of instrumental fertilization, and (ii) it may reduce the mutational load that is inflicted by oxidative stress on sperm^49^. Therefore, the use of oestrous stimulus mares during semen collection can have beneficial long-term consequences on the genetics of horse breeds. These insights could also be relevant for other mammals and in other contexts, for example in the management of captive populations or in supportive breeding programs^50^. Conservation programs for some endangered mammals would benefit from improved protocols of assisted reproductive techniques, for example, to minimize the loss of genetic diversity^51^ or to reduce mutational load^52^.

We conclude that sperm differ in their probability of fertilizing an ovum not only because of the female’s state (oestrus or dioestrus) but also because of the male’s reaction to the female’s state (increased quantities of seminal fluids and increased protection of sperm from oxidative stress). Our observations support predictions derived from ejaculate economics if the variation in female state that we studied here sufficiently represents variation in female fecundity as modelled by this theory^1^,^9^. This condition may only be fulfilled if the chance of fertilizing an ovum is not zero for sperm deposited in dioestrous females. Indeed, semen deposition and ovulation do not need to be synchronized for successful reproduction in many mammals^53^. Sperm storage is often possible in the isthmus and the oviduct. Sperm then bind to epithelial cells to form a reservoir that seems much under female control, as gene transcription and translation in these oviductal cells can support sperm storage and fertility^54^. One of the questions that remain to be answered is why, in response to the female’s cycle, males invest differentially into seminal fluids but not into sperm number per ejaculate.

## Additional Information

**How to cite this article:** Jeannerat, E. *et al*. Quality of seminal fluids varies with type of stimulus at ejaculation. *Sci. Rep.* **7**, 44339; doi: 10.1038/srep44339 (2017).

**Publisher's note:** Springer Nature remains neutral with regard to jurisdictional claims in published maps and institutional affiliations.

## Figures and Tables

**Table 1:** Effects of stallions’ and mares’ ID and oestrous stage on indicators of stallions’ sexual arousal and characteristics of fresh semen.

		Indicators of sexual arousal						Semen characteristics									
		Time to erection		Time to 1^st^ jump		Time to ejaculation		Sperm number		Sperm concentration		Semen volume		Sperm velocity^3^		Sperm motility	
(a) Multiple regressions																	
	d.f.	F	p	F	p	F	p	F	p	F	p	F	p	F	p	F	p
Stallion ID	15	9.3	**<0**.**001**	4.5	**<0**.**001**	3.7	**<0**.**001**	34.9	**<0**.**001**	20.5	**<0**.**001**	15.5	**<0**.**001**	11.4–28.4	**<0**.**001**	21.6	**<0**.**001**
Mare ID	1	0.03	0.86	1.7	0.20	1.4	0.23	0.05	0.82	<0.01	0.98	0.001	0.92	0.1–0.8	≥ 0.39	0.01	0.91
Oestrous stage^1^	2	5.6	**0**.**007**	3.7	**0**.**03**	3.8	**0**.**03**	0.61	0.55	3.2	**0**.**05**	2.6	**0**.**05**^2^	0.1–1.1	≥ 0.36	3.9	**0**.**03**
(b) Means ( ± 95% CI)																	
Dioestrus		55.6 ± 16.2 s		94.5 ± 11.0 s		130.4 ± 19.7 s		12.1 ± 2.4 × 10^9^		308 ± 60 × 10^6^/ml		43.6 ± 6.2 ml		120.2 ± 10.0 μm/s		58.9 ± 8.6%	
Oestrus		42.2 ± 15.4 s		91.1 ± 13.9 s		128.2 ± 34.0 s		11.7 ± 2.5 × 10^9^		282 ± 61 × 10^6^/ml		46.8 ± 8.3 ml		120.2 ± 9.7 μm/s		65.9 ± 9.3%	

(a) Results of multiple regression (bold font indicates statistical significance). (b) Means ( ± 95% confidence intervals) during dioestrus and oestrus.

^1^Oestrous or dioestrous, nested in mare ID; ^2^p_dir_; ^3^(a) range of the three velocity measures VAP, VSL, and VCL, (b) means (± 95% CI) for VAP.

**Table 2:** Effects of stallions’ and mares’ ID and oestrous stage on stallions’ plasma testosterone levels before and immediately after semen collection, and on sperm viability and lipid peroxidation 24 h and 48 h after ejaculation.

		Plasma testosterone around ejaculation (nmol/L)		Sperm viability (%)		Sperm lipid peroxidation (fluorescence intensity)	
(a) MANOVA:							
*Between-subjects:*	d.f.	F	p	F	p	F	p
Stallion ID	15, 45	9.2	**<0**.**001**	39.8	**<0**.**001**	2.0	**0**.**039**
Mare ID	1, 45	<0.01	0.98	1.0	0.33	0.7	0.41
Oestrous stage^1^	2, 45	3.0	0.06	1.5	0.23	7.8	**0**.**001**
* Within-subjects:*							
Time	1, 45	55.1	**<0**.**001**	18.9	**<0**.**001**	98.0	**<0**.**001**
Time × stallion	15, 45	1.8	0.07	0.9	0.53	1.3	0.22
Time × mares	1, 45	1.6	0.22	0.6	0.43	0.2	0.67
Time × oestrous stage^1^	2, 45	1.4	0.26	1.7	0.20	2.2	0.13
(b) Means ( ± 95% CI)^2^:							
Dioestrus (1^st^; 2^nd^ measurement)		3.7 ± 0.6; 4.2 ± 0.7		64.4 ± 7.8; 60.8 ± 7.8		63.3 ± 4.5; 78.9 ± 8.0	
Oestrus (1^st^; 2^nd^ measurement)		3.2 ± 0.6; 3.6 ± 0.6		66.1 ± 7.5; 64.6 ± 7.6;		53.8 ± 2.6; 64.7 ± 4.3	

(a) MANOVA (H-F corrected) on the two consecutive measurements each (bold font indicates statistical significance), and (b) means ( ± 95% confidence intervals) of the first and the second measurements for stallions exposed to dioestrous and oestrous mares.

^1^Oestrus or dioestrus, nested in mare ID; ^2^Means of the 32 pair combinations that were used for the MANOVA.

**Table 3:** Effects of stallions’ and mares’ ID and oestrous stage sperm velocity and motility 24 h and 48 h after ejaculation.

	VSL (μm/s)			VCL (μm/s)		VAP (μm/s)		Motility (%)	
(a) MANOVA:									
*Between-subjects:*	d.f.	F	p	F	p	F	p	F	p
Stallion ID	15, 45	10.7	**<0**.**001**	10.9	**<0**.**001**	11.6	**<0**.**001**	39.9	**<0**.**001**
Mare ID	1, 45	0.2	0.63	0.03	0.86	0.08	0.78	1.7	0.20
Oestrous stage^1^	2, 45	0.2	0.85	0.3	0.74	0.26	0.77	0.17	0.84
* Within-subjects:*									
Time	1, 45	1.0	0.31	12.2	**0**.**001**	8.6	**0**.**005**	51.0	**<0**.**001**
Time × stallion ID	15, 45	2.5	**0**.**009**	0.7	0.75	1.0	0.49	2.6	**0**.**006**
Time × mare ID	1, 45	1.6	0.21	4.5	**0**.**04**	2.0	0.16	0.5	0.48
Time × oestrous stage^1^	2, 45	1.1	0.36	0.6	0.53	0.8	0.45	0.3	0.73
(b) Means ( ± 95% CI)^2^:									
After 24 h		70.6 ± 5.4		167.2 ± 12.8		89.0 ± 7.2		57.8 ± 6.0	
After 48 h		71.6 ± 5.0		176.3 ± 12.0		92.9 ± 6.5		52.8 ± 6.2	

(a) MANOVA on the mean sperm velocity measures VSL (straight line velocity), VCL (curvilinear velocity), VAP (average path velocity), and total motility (% moving sperm) 24 h and 48 h after semen collection during exposure to mare A or B that was in oestrus or dioestrus (bold font indicates statistical significance), and (b) means ( ± 95% confidence intervals) of the first and the second measurements.

^1^Oestrus or dioestrus, nested in mare ID; ^2^Means of the 32 pair combinations tested in oestrus and dioestrus.
